# Supplementary material for: Content-rich biological network constructed by mining PubMed abstracts
Source: BMC Bioinformatics. 2004 Oct 8;5:147. doi: 10.1186/1471-2105-5-147 (PMC528731; doi:10.1186/1471-2105-5-147)
Supplement: Additional File 5 — The original Chilibot query results of the term "long-term potentiation (LTP)" and 22 other terms, limiting the latest references analyzed to the years 1990, 1995, 2000, and 2004. [file 1471-2105-5-147-S5.bz2 › chilibotAdditionalFile5/ltp2000/html/ERK_TAU.html]

 


 **ERK** and **TAU** 
  
Found 13 abstracts in PubMed,  **13 abstracts were retrieved and analyzed**.  


---

 Search Google  |
 PDF files only 
|  EDU domain only 

---

**Interactive relationship** (e.g. stimulation, inhibition, etc)

- The  **ERK**  and JNK cascades, which are downstream of Ras, have opposing effects on  **tau**  promoter activity  **ERK**  induces  **tau**  promoter activity, JNK inhibits it.  Ref: 9422391 J Neurochem, 1998
- **Tau**  promoter activity in PC12 cells is correlated with a short term activation of  **ERK** , which declines after a few hours and is followed by an activation of the inhibitory JNK cascade 76 h later.  Ref: 9422391 J Neurochem, 1998
- These observations suggest that the induction and inhibition of  **tau**  promoter are mediated by alternate  **ERK**  and JNK activities, which may underlie a mechanism to turn on and off genes during PC12 cell differentiation.  Ref: 9422391 J Neurochem, 1998
- A recently described protein kinase, PK40erk, 1 a member of the  **ERK**  family of kinases, can produce in vitro many of the properties of Alzheimer like hyperphosphorylated  **TAU** . cAMP dependent protein kinase A PKA phosphorylates  **TAU**  to a lesser extent.  Ref: 8166686 Biochem Biophys Res Commun, 1994
- Between 80% and 90% of IBM vacuolated muscle fibers contained well defined  **ERK**  immunoreactive inclusions, which were co localized by light microscopy, with phosphorylated  **tau**  in 70 to 80% of those fibers.  Ref: 10854206 Am J Pathol, 2000
- Finally, during a column fractionation and purification of  **Erk**  kinases from rat brain extracts, fractions enriched in  **Erk**  kinase activity also exhibited co elution of phosphorylated NF H, tubulin,  **tau**  and cdk5.  Ref: 10762698 Brain Res Mol Brain Res, 2000

**Parallel relationship** (e.g. studied together, co-existance, homology, etc.)

- The immunoreactivity of cortical and brainstem type Lewy bodies has been investigates with antibodies to the cyclin dependent kinase 5 cdk5, to the extracellular regulated kinase 1  **ERK**  1, and to the cdc2p34 kinase and with antibodies specific for phosphorylation epitopes typical of paired helical filament  **tau**  PHF  **tau** .  Ref: 7485409 Am J Pathol, 1995
- A possible link in support of this hypothesis is that one of the most striking features of AD, the abnormal accumulation of highly phosphorylated  **tau**  and neurofilament proteins, may be brought about by extracellular receptor kinase  **ERK**  whose activation is a common response to oxidative stress.  Ref: 10439473 Neuroreport, 1999
- Stress induced  **tau**  phosphorylation in mouse strains with different brain  **Erk**  1 2 immunoreactivity.  Ref: 9821159 Neurochem Res, 1998
- Both cortical and brainstem type Lewy bodies in diffuse Lewy body disease and brainstem type Lewy bodies in Parkinson s disease were found to be immunoreactive for cdk5 but not for cdc2p34 or  **ERK**  1 or with the PHF  **tau**  antibodies.  Ref: 7485409 Am J Pathol, 1995
- Our study suggests 1 that  **ERK** , a signal transducer, might play a role in IBM pathogenesis, including participation in the pathological phosphorylation of IBM  **tau** .  Ref: 10854206 Am J Pathol, 2000
